# Multiple Protein Kinases via Activation of Transcription Factors NF-κB, AP-1 and C/EBP-δ Regulate the IL-6/IL-8 Production by HIV-1 Vpr in Astrocytes

**DOI:** 10.1371/journal.pone.0135633

**Published:** 2015-08-13

**Authors:** Mohitkumar R. Gangwani, Anil Kumar

**Affiliations:** Division of Pharmacology and Toxicology, School of Pharmacy, University of Missouri, Kansas City, Missouri, United States of America; University of Nebraska Medical Center, UNITED STATES

## Abstract

Neurocognitive impairments affect a substantial population of HIV-1 infected individuals despite the success of anti-retroviral therapy in controlling viral replication. Astrocytes are emerging as a crucial cell type that might be playing a very important role in the persistence of neuroinflammation seen in patients suffering from HIV-1 associated neurocognitive disorders. HIV-1 viral proteins including Vpr exert neurotoxicity through direct and indirect mechanisms. Induction of IL-8 in microglial cells has been shown as one of the indirect mechanism through which Vpr reduces neuronal survival. We show that HIV-1 Vpr induces IL-6 and IL-8 in astrocytes in a time-dependent manner. Additional experiments utilizing chemical inhibitors and siRNA revealed that HIV-1 Vpr activates transcription factors NF-κB, AP-1 and C/EBP-δ via upstream protein kinases PI3K/Akt, p38-MAPK and Jnk-MAPK leading to the induction of IL-6 and IL-8 in astrocytes. We demonstrate that one of the mechanism for neuroinflammation seen in HIV-1 infected individuals involves induction of IL-6 and IL-8 by Vpr in astrocytes. Understanding the molecular pathways involved in the HIV-1 neuroinflammation would be helpful in the design of adjunct therapy to ameliorate some of the symptoms associated with HIV-1 neuropathogenesis.

## Introduction

HIV-1 associated neurocognitive disorders (HAND), which is attributed to the direct and indirect effects of HIV-1 penetration into the central nervous system (CNS) remains a major problem that adversely affects the quality of life in patients living with HIV-1 [1]. The introduction of Highly Active Anti-Retroviral Therapy (HAART) played a critical role in improving the quality of life and longevity of patients infected with HIV-1 [2]. Recent reports suggest that there is a decline in severe forms of dementia, however asymptomatic and minor cognitive disorders still affect close to 45% of HIV-1 infected individuals [3]. HIV-1 cannot directly infect neurons so it is believed that infection of other major cell types present in the brain such as microglia and astrocytes, and subsequent release of neurotoxins is responsible for the neuronal damage and ultimately their demise [1,4]. Neurotoxins such as viral proteins or pro-inflammatory cytokines have been shown in a variety of studies to have a profound effect on the viability of the neurons [5,6]. Apart from the entire virus, the associated viral proteins have been postulated to play a crucial role in formation of neuroinflammatory milieu in CNS [7]. These cytokines are secreted to combat the pathological insult, however because of their ability to communicate and co-operate with other host and viral mediators, they can generate and intensify undesirable effects. Furthermore, presence of cytokines such as IFN-γ helps the virus in productively infecting astrocytes that will otherwise show restricted infection [8].

HIV-1 encoded accessory gene product Vpr (Viral Protein R) performs several critical functions in the viral life cycle, including the transport of HIV-1 pre-integration complex into the nucleus of infected cells [9]. Vpr has been shown in the brain sections of HIV-1 encephalitis patients [10] and also induces neuronal apoptosis via direct and indirect mechanisms [6,11]. It was found that one of the indirect mechanisms through which Vpr promotes neuronal death involves signaling through IL-1β and IL-8 [6]. IL-8 has been shown in other studies as well to upregulate proteins involved in neuronal apoptosis and cause neuronal cell death [12]. HIV-1 Vpr has increased the expression of IL-8 and IL-6 in U937 monocytes [13]. Moreover, Vpr mediated production of IL-6 induces reactivation of HIV-1 production in latently infected monocytes/macrophages [14]. Chronic overexpression of IL-6 in the brains of transgenic mice produces neurologic disease with symptoms of neuronal cell degeneration [15]. All these studies suggest that IL-6 and IL-8 could be the mediators of neuroinflammation seen in HIV-1 infected individuals, which is implicated in the development HAND.

Astrocytes are the most abundant cell type in the brain. They serve many critical physiological roles in the brain and thus are responsible for maintaining the homeostasis. It is becoming clear that astrocytes play a crucial role in the development of HAND [16]. Perivascular astrocytes are closely involved in the maintenance of Blood brain barrier (BBB) integrity, and their dysregulation could lead to increased HIV-1 entry into the brain with enormous consequences [17,18]. The abundance of astrocytes and their ability to produce a multitude of cytokines and chemokines in response to pathogenic insult make them a suitable target to investigate the molecular mechanisms necessary to promote the induction of neuroinflammatory molecules in response to HIV-1 Vpr [19]. Treatment of primary human fetal astrocytes with exogenous HIV-1 Vpr protein induces IL-6 and IL-8 secretion, as evidenced in a recent study [20]. In this study, our major goal was to elucidate the mechanism(s) responsible for the induction of IL-6 and IL-8 by HIV-1 Vpr in astrocytes. Understanding these mechanisms would ultimately help in the design of adjunct therapies to alleviate the symptoms of HAND.

## Materials and Methods

### Cell culture and reagents

Human fetal astrocytic cell line, SVGA was kindly provided by Dr. Avindra Nath [21]. These cells were cultured in Dulbecco’s Modified Eagle Medium (DMEM, Cellgro) which was supplemented with 10% Fetal Bovine Serum, 1% L-glutamine, 1% Non-essential amino acids, 1% sodium bicarbonate and 50 μg/ml of gentamycin. The cells were maintained in a humidified chamber at 37°C and 5% CO_2_ environment. Lipofectamine 2000 was purchased from Invitrogen Inc. (Carlsbad, CA). Chemical inhibitors for NF-κB pathway (SC514, Bay 11–7082), p38-MAPK (SB203580), PI3K/Akt (LY294002), Erk-MAPK (UO126) and Jnk-MAPK (SP600125) were obtained from Cayman Chemicals (Ann Arbor, Michigan, USA). Pre-designed siRNA’s against NF-κB pathway [p50 (NFΚB1), p65 (RelA)], p38-MAPK isoforms (p38α, p38β, p38γ, p38δ), Akt isoforms (Akt-1, Akt-2, Akt-3), C/EBP isoforms (C/EBP-β, C/EBP-δ) and AP-1 were purchased from Thermo Fisher Scientific Inc. (Waltham, MA). Construction of HIV-Vpr plasmid has been previously described [22].

### Isolation and cultivation of primary human fetal astrocytes

Human astrocytes were isolated from elective aborted brain specimens obtained from the Birth Defects Laboratory (University of Washington, Seattle) in full compliance with the ethical guidelines of the National Institutes of Health (NIH), University of Washington, and university of Missouri-Kansas City as previously described [23]. Briefly, the brain tissues were dissected and mechanically dissociated using 25 ml pipettes. The tissue was then washed and passed through a sterile nylon mesh with 250μm pore size. The cells were then passed through a 70 mM strainer (Corning Inc., Tewksbury, MA, cat # 3520350) into 50 ml tube. The cell suspension was then centrifuged and suspended in astrocyte medium (DMEM with F12 nutrient mixture, L-glutamine and HEPES (Life Technologies, cat # 14175079), 10% FBS, 0.5 ml gentamycin) and plated at the density of 20×10^6^ cells/150 cm^2^. The astrocytes were cultured to increase the purity and they were routinely >99% pure as determined by staining for glial fibrillary acidic protein (GFAP).

### Transfection

Lipofectamine 2000 was used to transiently transfect SVGA astrocytes as per the previously optimized protocol recommended by manufacturer. Briefly, 0.275 × 10^6^ astrocytes adhered per well of 12-well plate were washed with PBS (phosphate buffered saline) and supplied with serum and antibiotic free DMEM. SVGA astrocytes were incubated with a transfection mixture containing 0.5 μg of HIV-1 Vpr plasmid and 2 μl of lipofectamine in a low serum medium (Opti-MEM, Life technologies). After 5h of incubation, the cells were washed with PBS and provided with serum and antibiotic containing DMEM. The inhibitor experiments were carried out by 1h pre-treating the astrocytes with chemical inhibitors for various pathways and then transfecting them with HIV-1 Vpr encoding plasmid. Mock-transfected astrocytes were used as a control. For siRNA knockdown experiments, the cells were transfected for 24h in serum and antibiotic free medium with siRNAs followed by 24h of culturing in complete DMEM. The astrocytes were then either mock-transfected or were transfected with a plasmid encoding HIV-1 Vpr. The mRNA expression levels of IL-6 and IL-8 were quantified at 6h post transfection, while IL-6 and IL-8 proteins were determined after 48h of transfection. Electroporation was carried out in primary human fetal astrocytes with Amaxa Basic Glial Cells Nucleofector Kit (Lonza, cat # VPI-1006) using the manufacturers recommendations. Briefly, 2 μg of Vpr plasmid was mixed with the transfection mixture and added to 8×10^6^ astrocytes, mixed gently and transferred to electrode tubes for electroporation. The tubes were removed quickly after electroporation and complete DMEM was added very slowly to the cells, followed by transferring the cells to the 6 well plates. The cells were harvested after 24h of electroporation. The transfection efficiency was found between 15–18% using flow cytometer.

### Real time RT-PCR

RNeasy mini kits (Qiagen, Valencia, CA) were used to extract total RNA as per the manufacturer’s protocol. The extracted RNA was amplified in Bio-Rad iCycler iQ5 using commercially synthesized primer sequences for IL-6 and IL-8 with previously published PCR conditions [24,25]. 2^-ΔΔCt^ method was utilized to calculate the relative expression levels of IL-6 and IL-8 using HPRT (hypoxanthine-guanine phosphoribosyl transferase) as a house keeping control.

### BioPlex multicytokine assay

For the determination of IL-6 and IL-8 protein concentration in cell culture supernatants, multiple cytokine assay kits (Bio-Rad, Hercules; CA) were utilized. Briefly, the supernatants were cleared by removing cell debris through centrifugation (5000 rpm for 5 min– 2×) and allowed to react with magnetic beads coated with IL-6 and IL-8 antibodies for 30 min. The plates were then probed with detection antibody for 30 min, followed by incubation in streptavidin-PE for 10 min. The beads were washed and re-suspended in assay buffer and the concentration was determined using 5PL standard curve method in Bio-Plex manager 5.0 software.

### Immunocytochemistry

6 × 10^5^ astrocytes were seeded in glass cover slips in 6-well plates and allowed to adhere overnight. The cells were then transfected with HIV-1 Vpr encoding plasmid for 5h as detailed in transfection section. The transfection medium was replaced with complete DMEM with 1 mg/ml GolgiStop (BD Biosciences, San Jose, CA) to prevent the release of IL-6 and IL-8. At the end of 6h, the astrocytes were fixed with ice-cold methanol and acetone (1:1) solution for 20 min at 20°C. The cells were washed 3× with PBS and permealized with PBST (PBS containing 0.1% TritonX-100). Blocking was carried out using 1% BSA (Bovine Serum Albumin) in PBST for 30 min at room temperature. The cells were incubated overnight with primary antibodies against IL-6, IL-8 and GFAP (Abcam, Inc.) in a humidified chamber. The cells were washed 5x with PBST and incubated for 1h in secondary antibodies conjugated with Alexa Fluor 488 (Anti-Rabbit, 1:1000; Cell Signaling Technology) and Alexa Fluor 555 (Anti-Mouse, 1:1000; Cell Signaling Technology) at room temperature in the dark. All the antibodies were diluted in PBS containing 1% BSA. The glass cover slips with immunostained astrocytes were mounted on glass slides with 10 μl of vectashield mounting medium containing DAPI (Vector Laboratories, Burlingame, CA). The cover slips were made immovable using nail polish without toluene. The images were captured using Leica TCS SP5 II (Leica Microsystems, Wetzler, Germany) on an inverted microscope platform with 40x zoom lens. The intensity of images was calculated using mutimeasure tool from NIH ImageJ software. The intensity values for IL-6 and IL-8 were normalized to astrocyte marker GFAP.

### Western blotting

SVGA astrocytes were either mock-transfected or were transfected with a plasmid encoding HIV-1 Vpr and they were harvested after indicated times, followed by the separation of nuclear and cytosolic extracts using NE-PER nuclear extraction kit (Pierce, Rockford, IL). Total cell extracts were prepared by lysing cells after transfection using RIPA buffer (Boston Bioproducts, Ashland, MA). The lysates were homogenized for 15s and centrifuged at 14000 × g for 15 min to eliminate cell debris. Protein concentrations were determined using BCA protein assay kit (Pierce, Rockford, IL) and 20 μg of protein was resolved on 10% SDS-PAGE (75 V for 3h). For immunoblotting, proteins were electrophoretically transferred (350 mA for 1.5h) onto a PVDF membrane and blocked overnight at 4°C with 5% nonfat milk in PBST (PBS with 0.075% Tween-20). All the primary antibodies used were purchased from cell signaling (Cell Signaling technology, Inc.) and used at 1:2000 dilution except p-p38 (Santa Cruz Biotechnology, Inc.– 1:1000). The membrane was washed 5× with PBST and was incubated for 2h at room temperature in secondary antibodies conjugated to horseradish peroxide. The membrane was washed 5× and the bands were visualized using BM chemiluminiscence western blotting substrate (POD, Roche Applied Sciences; Indianapolis, IN). FluorChem HSD2 software (Alpha Innotech, San Leandro, CA) was used for the quantification of the bands.

### Statistical analyses

The results are expressed as mean ± SEM (Standard error of mean) of 3 independent experiments and each experiment was performed in triplicates. The statistical analyses for multiple comparisons were performed using 1-way ANOVA followed by Tukey honestly significant difference (HSD) post hoc test. All the statistical analyses were conducted using SPSS (IBM Corp. Released 2012. IBM SPSS Statistics for Windows, Version 21.0. Armonk, NY: IBM Corp) and the results were considered statistically significant at p value ≤ 0.05.

## Results

### HIV-1 Vpr time-dependently induces the production of IL-6 and IL-8 in astrocytes

**Fig 1:**
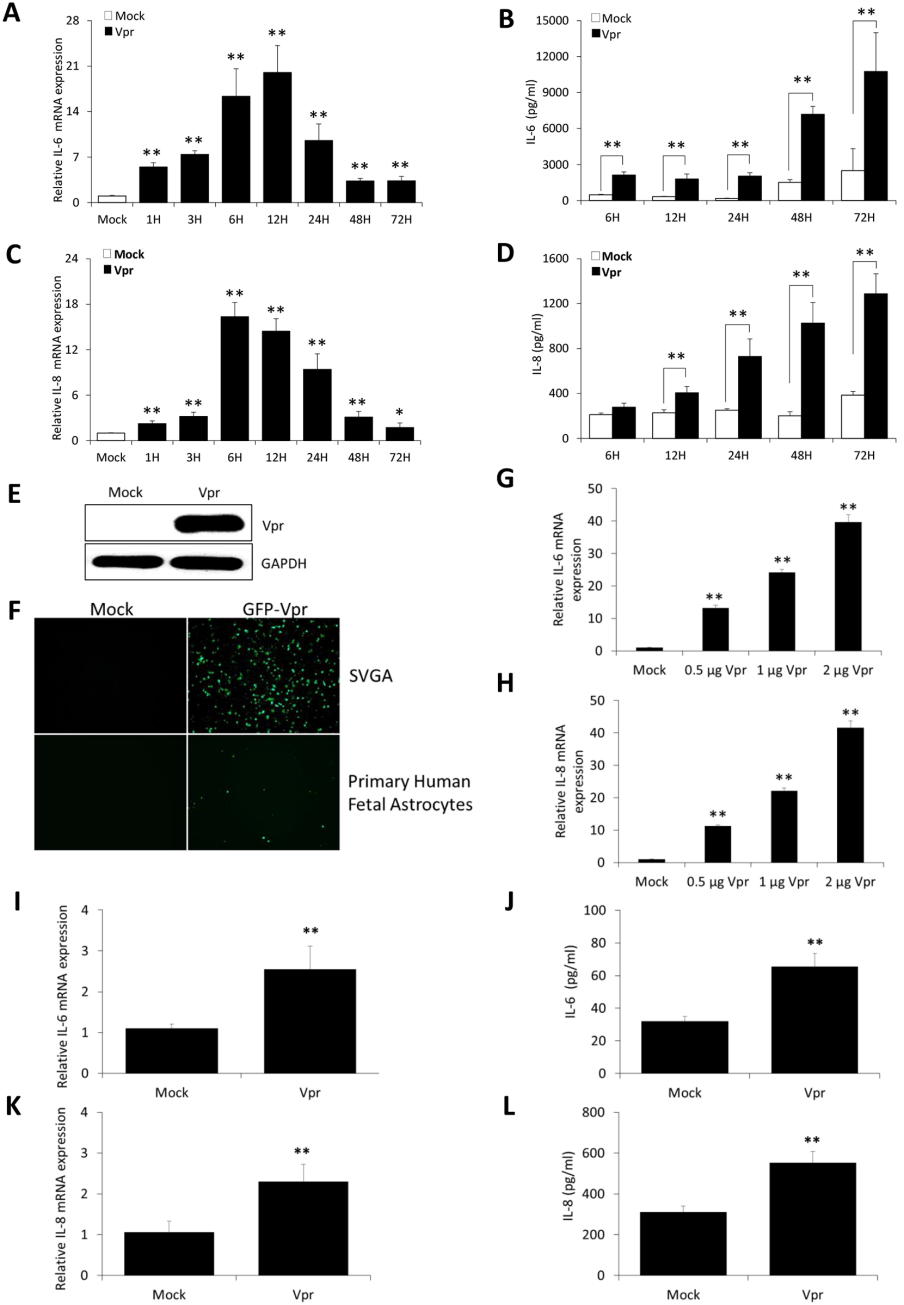
HIV-1 Vpr time-dependently induces IL-6 and IL-8 in astrocytes. SVGA astrocytes were seeded in 6 well plates and transfected with a plasmid encoding HIV-1 Vpr or were mock-transfected. The cells were harvested at 1, 3, 6, 12, 24, 48 and 72h post-transfection followed by the determination of IL-6 and IL-8 mRNA expression levels using real-time RT-PCR. The cell culture supernatants were also collected at 6, 12, 24, 48 and 72h post-transfection, and protein concentration of IL-6 and IL-8 was determined using BioPlex multi-cytokine assay. **(A, C)** mRNA expression levels of IL-6 and IL-8 calculated relative to mock-transfected controls, respectively. **(B, D)** protein concentration for secreted IL-6 and IL-8 in cell culture supernatants, respectively. **(E)** Expression of Vpr in SVGA astrocytes using western blotting. **(F)** Expression of GFP-Vpr in SVGA and primary astrocytes using fluorescent microscopy. **(G, H)** dose-dependent induction of IL-6 and IL-8 by HIV-1 Vpr plasmid in SVGA astrocytes. **(I, J)** depicts the mRNA expression levels, while **(K, L)** shows the secreted protein levels of IL-6 and IL-8 in human fetal astrocytes by HIV-1 Vpr electroporation, respectively. Every bar represents the mean ± SE of three independent experiments done in triplicates. Statistical analyses were performed using 1-way ANOVA using post-hoc Tukey HSD test, ** p < 0.01 and * p < 0.05.

Neurotoxic potential of IL-6 and IL-8 is very well documented in various neurodegenerative diseases including HAND. HIV-1 Vpr has been shown to induce IL-6 in macrophages and IL-8 in T-cells and monocytes/macrophages [13]. However, it is not known if Vpr has similar tendency in astrocytes. To this end, SVGA astrocytes were transfected with a plasmid encoding for HIV-1 Vpr, and the cells were harvested at 1, 3, 6, 12, 24, 48 and 72h post transfection to determine the mRNA expression levels of IL-6 and IL-8. Cell culture supernatants were also collected after 6, 12, 24, 48 and 72h of transfection to determine the levels of secreted IL-6 and IL-8. Astrocytes were transfected with GFP (Green Fluorescence Protein) encoding plasmid in parallel to get an estimation about the transfection efficiency. We routinely got transfection efficiency in the range of 55–70% (data not shown) as determined using flow cytometer (BD FACScanto II). The expression of HIV-1 Vpr in the astrocytes was confirmed by transfection of cells with GFP-Vpr followed by western blotting and fluorescent microscopy (Fig 1E and 1F). We observed a time dependent increase in the production of IL-6 (Fig 1A and 1B) and IL-8 (Fig 1C and 1D). The peak IL-6 and IL-8 mRNA was found at 12h (20.03 ± 4.13 Fold) and 6h (16.36 ± 1.86 Fold) post transfection as compared to mock-transfected controls, respectively. The secreted IL-6 and IL-8 protein levels were increased at all-time points assayed with a peak at 72h (10,762 ± 3230 pg/ml; 1287 ± 178 pg/ml), as compared to mock-transfected controls (2495 ± 1826 pg/ml; 385 ± 34 pg/ml), respectively. We also observed an increase in protein levels of mock-transfected cells at 72h which could be due to an increase in the number of cells with time in the plate. We also observed a Vpr plasmid dose-dependent induction of IL-6 and IL-8 in astrocytes (Fig 1G and 1H). We have used the dose of 0.5 μg Vpr in our studies. We further confirmed the increased expression of IL-6 (Fig 1I and 1J) and IL-8 (Fig 1K and 1L) in primary human fetal astrocytes by electroporation of plasmid encoding HIV-1 Vpr. We did not see induction of IL-6 and IL-8 by HIV-1 Vpr in every donor. Astrocytes from 4 of 6 donors showed significant IL-6 and IL-8 upregulation at both RNA and protein levels. Whereas donor-2 showed slight increase that was statistically not significant. The 6^th^ donor showed no IL-6 or IL-8 upregulation. Expression of IL-6 and IL-8 in individual donors is shown in S1 Fig.

### Immunostaining for HIV-1 Vpr mediated production of IL-6 and IL-8 in SVGA astrocytes

**Fig 2:**
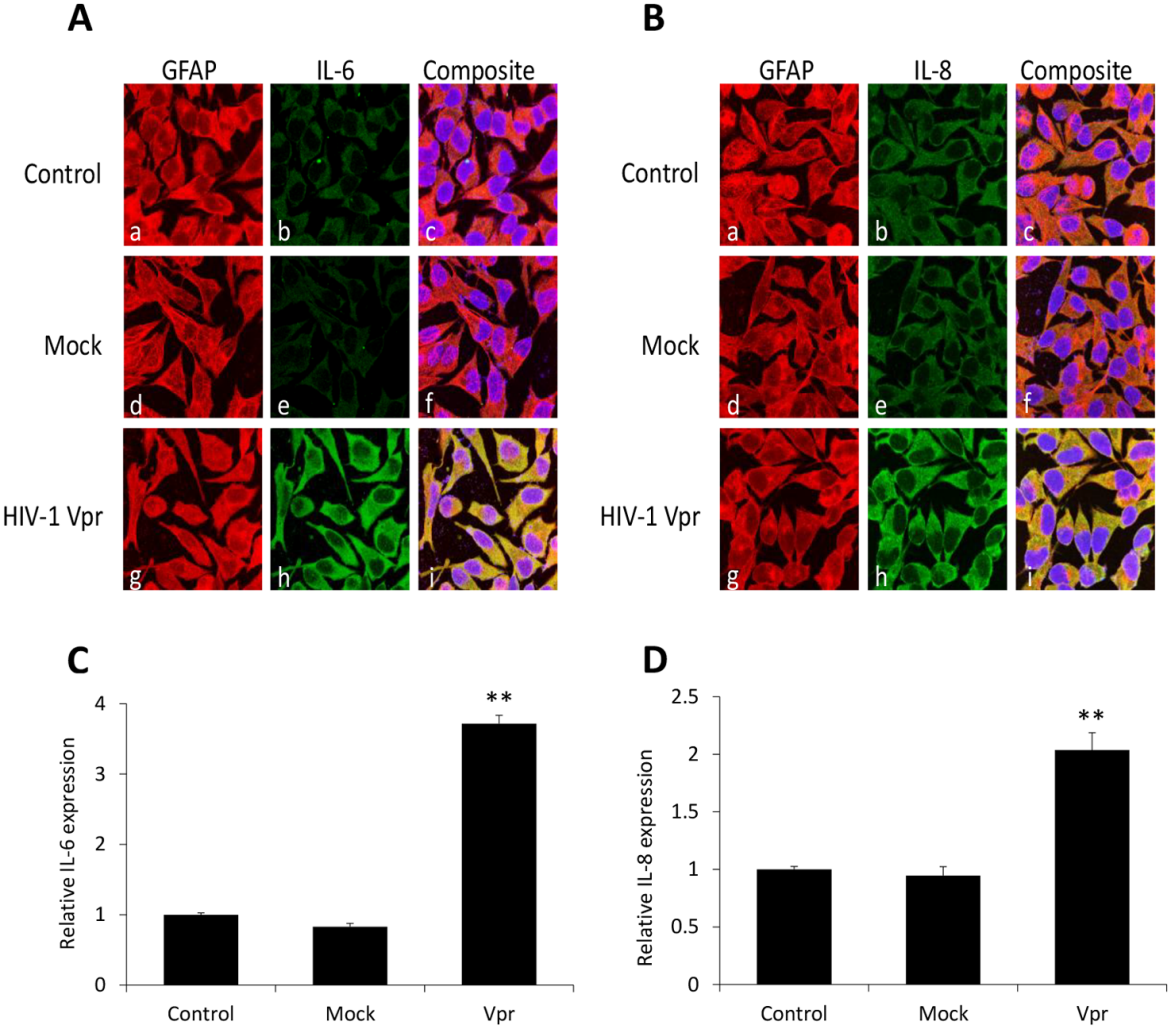
Immunocytochemical staining portraying induction of IL-6 and IL-8 by HIV-1 Vpr in SVGA astrocytes. SVGA astrocytes were cultured on cover slips and were either mock-transfected **(d-f)** or were transfected with a plasmid encoding Vpr **(g-i)**. Non-transfected cells were used as control **(a-c)**. The cells were stained for nucleus (*blue*); IL-6 and IL-8 (*green*) and GFAP (*red*). The images were captured using a Leica TCS SP5 II on an inverted microscope platform with a 40× zoom oil emersion lens. The Image J software was used to get the merged images, and the Multi Measure tool in Image J was used to quantify the intensities. **(A, B)** Immunocytochemical staining depicting induction of IL-6 and IL-8 by HIV-1 Vpr in SVGA astrocytes. **(C, D)** The image intensities for IL-6 and IL-8 are plotted relative to GFAP. Every bar represents the mean ± SE of thee independent experiments done in triplicates. Statistical analyses were performed using 1-way ANOVA using post-hoc Tukey HSD test, ** p < 0.01 and * p < 0.05.

To further demonstrate the induction of IL-6 and IL-8 by HIV-1 Vpr in astrocytes, we performed immunocytochemistry on HIV-1 Vpr transfected astrocytes. After 5h of transfection, the release of IL-6 and IL-8 was prevented using GolgiStop treatment for 6h and the astrocytes were immunostained with antibodies for IL-6, IL-8 and GFAP. Visualization of these proteins was then carried out using secondary antibodies displaying green (IL-6 and IL-8) and red (GFAP) colors. The nuclei were stained with DAPI (Blue). HIV-1 Vpr transfected astrocytes showed a strong yellow signal in the composite images indicating the accumulation of IL-6 (Fig 2Ai) and IL-8 (Fig 2Bi) which was colocalized with GFAP as compared to untransfected (Fig 2Ac and 2Bc) or mock-transfected (Fig 2Ag and 2Bg) astrocytes, respectively. Quantification of images using NIH ImageJ software confirmed statistically significant increases in IL-6 (Fig 2C) and IL-8 (Fig 2D) levels in HIV-1 Vpr transfected astrocytes when compared with untransfected or mock-transfected controls. The bars display the intensities of IL-6 and IL-8 normalized to GFAP as a control.

### HIV-1 Vpr increases IL-6 and IL-8 in astrocytes through NF-κB pathway

**Fig 3:**
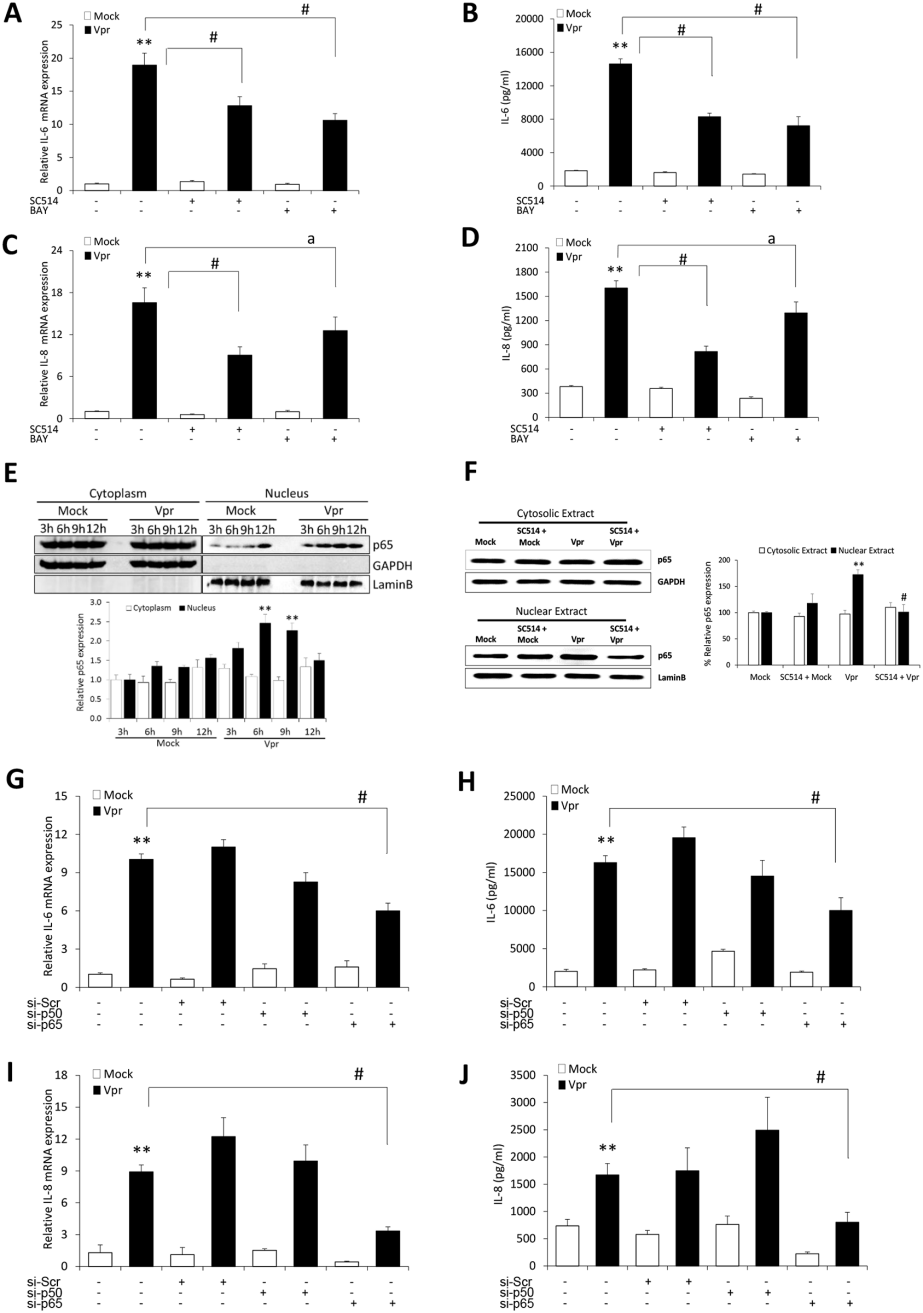
HIV-1 Vpr utilizes NF-κB pathway to induce IL-6 and IL-8 in SVGA astrocytes. SVGA astrocytes were allowed to adhere overnight in 6 well plates. The cells were then 1h pre-treated with chemical inhibitor of NF-κB pathway (SC514 or BAY 1170–82) and then transfected with a plasmid encoding HIV-1 Vpr or were mock-transfected. The cells were harvested at 6h post-transfection followed by the determination of IL-6 and IL-8 mRNA expression levels using real-time RT-PCR. The cell culture supernatants were also collected at 48h post-transfection, and protein concentration of IL-6 and IL-8 was determined using BioPlex multi-cytokine assay. **(A, C)** mRNA expression levels of IL-6 and IL-8 calculated relative to mock-transfected controls in the presence of chemical inhibitors, respectively. **(B, D)** protein concentrations for secreted IL-6 and IL-8 in the presence of chemical inhibitor, respectively. **(E)** Time kinetics for NF-κB p-65 nuclear translocation in response to HIV-1 Vpr **(F)** depicts the effect of SC514 on NF-κB p-65 nuclear translocation. **(G, I)** portray mRNA levels while **(H, J)** show protein concentration for IL-6 and IL-8 when NF-κB p50 and p65 subunits were silenced. Every bar represents the mean ± SE of thee independent experiments done in triplicates. Statistical analyses were performed using 1-way ANOVA using post-hoc Tukey HSD test, **#** p < 0.01, ^**a**^ p < 0.05 as compared to Vpr transfected cells; ** p < 0.01 and * p < 0.05 compared to mock-transfected controls.

NF-κB is a master regulator of a myriad of inflammatory genes. To understand the role of NF-κB in the elevation of IL-6 and IL-8 by HIV-1 Vpr, we pre-treated the cells with chemical inhibitors for NF-κB (SC514, BAY 11–7082) pathway. Optimum dose of the inhibitor was decided based on its IC50 values and the effects on cell viability. The inhibitor was added 1h before transfection and was present throughout the experiment. SC514 and BAY 1170–82 (Fig 3A and 3C) significantly reduced the mRNA expression of IL-6 (32.4 ± 7.2%; 45.7 ± 6.9%) and IL-8 (43.9 ± 5.2%; 29.8 ± 11.8%) as compared to untreated controls. We observed similar reductions with SC514 and BAY 1170–82 (Fig 3B and 3D) in the levels of secreted IL-6 (43.3 ± 4.8%; 50.5 ± 8.2%) and IL-8 (50.8 ± 3.6%; 23.7 ± 7.6%) proteins, respectively. NF-κB is known to translocate from cytoplasm to nucleus in response to stimulus and thus we performed western blotting to estimate the levels of nuclear translocation of NF-κB with HIV-1 Vpr. As shown in the figure (Fig 3E), at 6h and 9h there was substantial increase in the p65 levels in nuclear extracts of HIV-1 Vpr transfected as compared to mock-transfected astrocytes. This elevated NF-κB translocation was brought back to nearly the mock levels with the pre-treatment of SC514 (Fig 3F), which corroborates the involvement of NF-κB in HIV-1 Vpr mediated induction of IL-6 and IL-8 in astrocytes. The role of NF-κB in HIV-1 Vpr mediated induction of IL-6 and IL-8 in astrocytes was further confirmed by the use of specific siRNAs directed against p50 and p65 subunits of NF-κB. Knockdown of p65 subunit (Fig 3G–3J) substantially reduced the mRNA and protein expression levels of IL-6 (42.7 ± 5.3%; 42.5 ± 10.2%) and IL-8 (62.6 ± 4.4%; 52.1 ± 10.8%), respectively. While knockdown of p50 showed slight but statistically insignificant inhibition in IL-6 expression, it showed increase in IL-8 levels which might be due to the removal of repressive effects of p50 homodimers on IL-8 promoter by p50 knockdown [26].

### PI3K/Akt mediated activation of NF-κB is involved in HIV-1 Vpr induced upregulation of IL-6 and IL-8 in astrocytes

**Fig 4:**
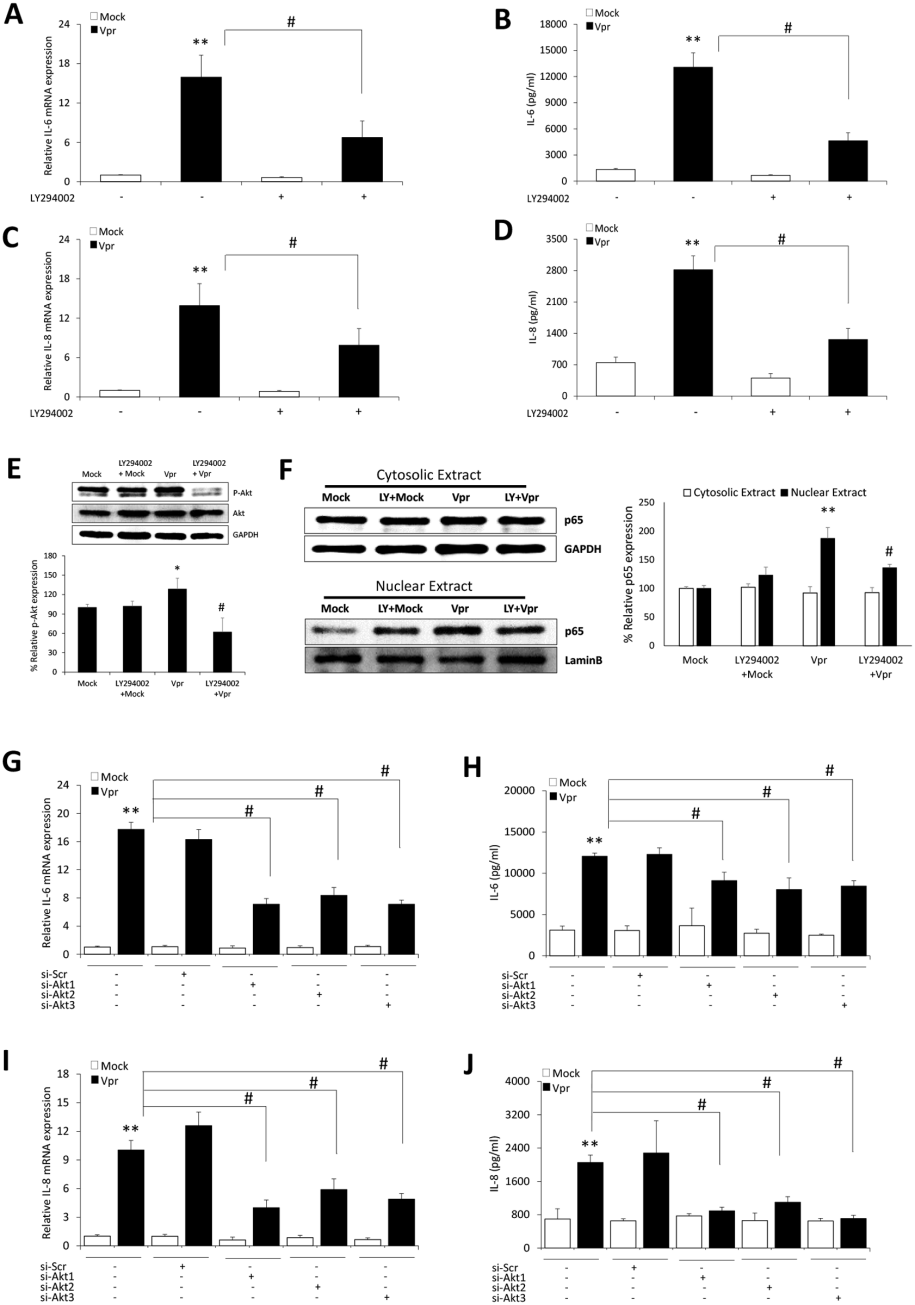
HIV-1 Vpr mediated induction of IL-6 and IL-8 in astrocytes involves PI3K/Akt pathway. SVGA astrocytes were cultured and seeded in 6 well plates. The cells were 1h pre-treated with chemical inhibitor for PI3K/Akt pathway (LY294002) and then transfected with a plasmid encoding HIV-1 Vpr or were mock-transfected. The cells were harvested at 6h post-transfection followed by the determination of IL-6 and IL-8 mRNA expression levels using real-time RT-PCR. The cell culture supernatants were collected at 48 h post-transfection, and protein concentration of secreted IL-6 and IL-8 was determined using BioPlex multi-cytokine assay. **(A, C)** mRNA expression levels of IL-6 and IL-8 calculated relative to mock-transfected controls in the presence of LY294002, respectively. **(B, D)** Protein concentrations for secreted IL-6 and IL-8 with LY294002, respectively. **(E, F)** Depicts the effect of LY294002 on phosphorylated Akt and NF-κB p-65 nuclear translocation, respectively. **(G, I)** portray mRNA levels while **(H, J)** show protein concentration for IL-6 and IL-8 when individuals Akt isoforms were silenced, respectively. Every bar represents the mean ± SE of thee independent experiments done in triplicates. Statistical analyses were performed using 1-way ANOVA using post-hoc Tukey HSD test, **#** p < 0.01, ^**a**^ p < 0.05 as compared to Vpr transfected cells; ** p < 0.01 and * p < 0.05 compared to mock-transfected controls.

PI3K/Akt is well established as one of the upstream mechanisms responsible for the activation of NF-κB signaling pathway. We also have recently shown that PI3K/Akt mediated activation of NF-κB is involved in HIV-1 Vpr mediated induction of CCL5 in astrocytes [22]. To identify the role of PI3K/Akt signaling in upregulation of IL-6 and IL-8 through NF-κB pathway, we employed LY294002 (PI3K/Akt inhibitor) before transfection with the plasmid encoding HIV-1 Vpr. Pre-treatment with LY294002 abrogated the mRNA expression levels of IL-6 and IL-8 by 57.8 ± 15.8% and 43.4 ± 11.1%, respectively (Fig 4A and 4C). We also observed statistically significant reduction in the secreted IL-6 (64.6 ± 7.1%) and IL-8 (55.3 ± 8.8%) protein levels with LY294002 pre-treatment in HIV-1 Vpr transfected astrocytes as compared to untreated cells (Fig 4B and 4D). LY294002 has been shown to target other signaling molecules apart from PI3K/Akt pathway. Hence the effect of LY294002 on the levels of phosphorylated Akt and p65 nuclear translocation was tested in HIV-1 Vpr transfected astrocytes. We observed a significant reduction in the levels of phosphorylated Akt (Fig 4E) and p65 nuclear translocation (Fig 4F) in the presence of LY294002 with HIV-1 Vpr in astrocytes. To further confirm the role of PI3K/Akt pathway, we employed specific siRNA’s to knockdown individual Akt isoforms. The silencing of all the Akt isoforms was able to substantially reduce the mRNA (Fig 4G and 4I) and secreted protein levels (Fig 4H and 4J) of IL-6 and IL-8, respectively.

### Role of p38 and Jnk MAP kinases in HIV-1 Vpr mediated induction of IL-6 and IL-8 in astrocytes

**Fig 5:**
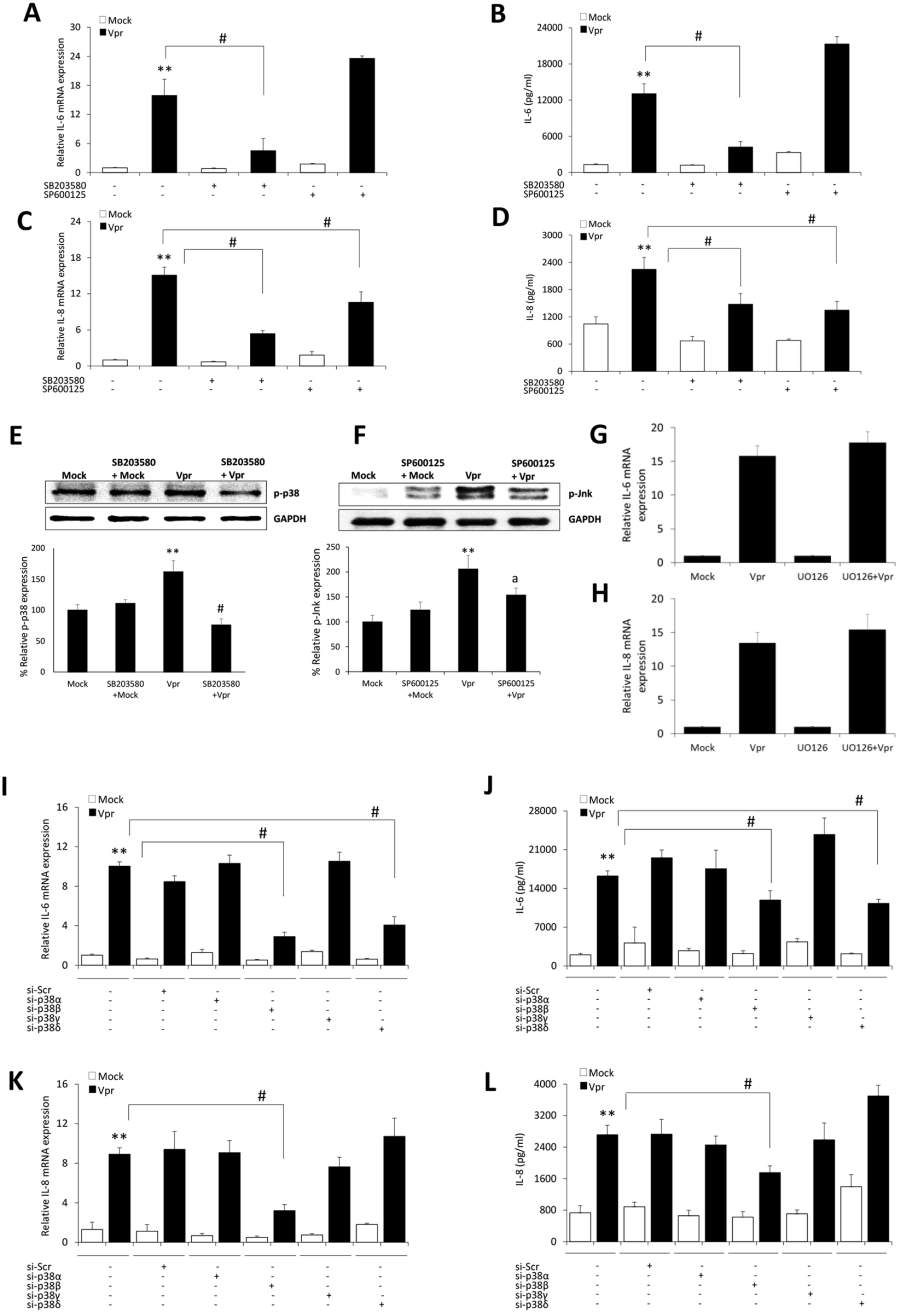
HIV-1 Vpr mediated induction of IL-6 and IL-8 in astrocytes involves MAPK pathway. SVGA astrocytes were cultured and seeded in 6 well plates. The cells were 1h pre-treated with chemical inhibitor for MAPK pathway (SB203580 –p38; SP600125 –Jnk and UO126—Erk) and then transfected with a plasmid encoding HIV-1 Vpr or were mock-transfected. The cells were harvested at 6h post-transfection followed by the determination of IL-6 and IL-8 mRNA expression levels using real-time RT-PCR. The cell culture supernatants were collected at 48h post-transfection, and protein concentration of secreted IL-6 and IL-8 was determined using BioPlex multi-cytokine assay. **(A, C)** mRNA expression levels of IL-6 and IL-8 calculated relative to mock-transfected controls in the presence of chemical inhibitors, respectively. **(B, D)** Protein concentrations for secreted IL-6 and IL-8 with chemical inhibitors, respectively. **(E, F)** Depicts the effect of SB203580 and SP600125 on phosphorylated p38 and Jnk MAPK’s, respectively. **(G, H)** represents the effect of Erk-MAPK inhibitor UO126 on the mRNA expression levels of IL-6 and IL-8, respectively. **(I, K)** portray mRNA levels while **(J, L)** show protein concentration for IL-6 and IL-8 when individuals p38-MAPK isoforms were silenced, respectively. Every bar represents the mean ± SE of thee independent experiments done in triplicates. Statistical analyses were performed using 1-way ANOVA using post-hoc Tukey HSD test, **#** p < 0.01, ^**a**^ p < 0.05 as compared to Vpr transfected cells; ** p < 0.01 and * p < 0.05 compared to mock-transfected controls.

Mitogen activated protein kinases (MAPK’s) have been extensively linked to the regulation of expression of inflammatory genes. We have also shown in our previous studies that induction of IL-6 and IL-8 is regulated by p38 MAPK. We wanted to test the possibility of involvement of MAPK pathways in the induction of IL-6 and IL-8 by HIV-1 Vpr in astrocytes. We pre-treated the cells with optimum dose of chemical inhibitors targeting p38 (SB203580), Erk (UO126) and Jnk (SP600125) pathways. The cells were then mock-transfected or were transfected with a plasmid encoding HIV-1 Vpr. We observed a substantial abrogation in the mRNA levels of IL-6 (71.6 ± 3.3%) and IL-8 (64.4 ± 4.2%) by SB203580 (Fig 5A and 5C). We observed a similar trend in the secreted IL-6 and IL-8 protein levels by SB203580 with the reductions of 67.7 ± 9.6% and 38.3 ± 4.2%, respectively (Fig 5B and 5D). On the other hand, SP600125 only showed inhibition in the mRNA and protein levels of IL-8 by 29.8 ± 6.2% and 40.2 ± 9.3%, respectively (Fig 5A–5D). We found no inhibition in the levels of IL-6 and IL-8 levels with UO126 (Fig 5G and 5H). The off-target effects of chemical inhibitors were ruled out by quantifying the phosphorylated forms of p38 and Jnk following HIV-1 Vpr transfection in the presence SB203580 and SP600125, respectively (Fig 5E and 5F). It is known that SB203580 has more specific targeting effect on p38α and p38β isoforms with negligible effect on p38γ and p38δ isoforms [27]. Therefore, we tested siRNA’s directed towards individual p38 MAPK isoforms. We observed reduction in the mRNA and secreted protein levels of IL-6 and IL-8 by p38β MAPK isoform silencing, however p38δ isoform silencing was only able to reduce the mRNA and protein levels of IL-6 (Fig 5I–5L).

### Involvement of C/EBP-δ and AP-1 transcription factors in HIV-1 Vpr mediated increased expression of IL-6 and IL-8 in astrocytes

**Fig 6:**
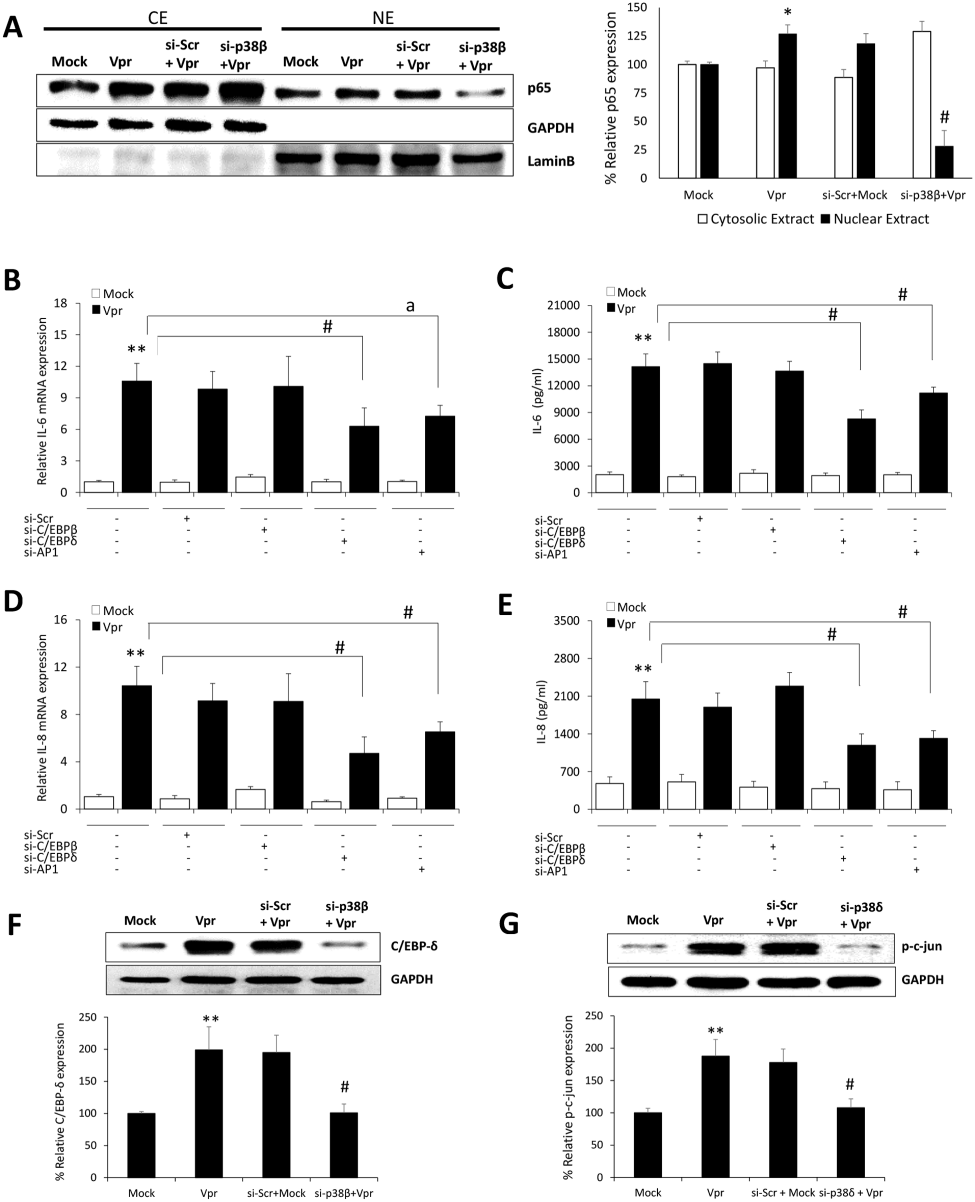
HIV-1 Vpr involves C/EBP-δ and AP-1 transcription factors in the induction of IL-6 and IL-8 in astrocytes . SVGA astrocytes were cultured and seeded in 6 well plates. The cells were transfected with siRNA against C/EBP isoforms and AP-1 for 48h. Then the cells were transfected with a plasmid encoding HIV-1 Vpr or were mock-transfected. The cells were harvested at 6h post-transfection followed by the determination of IL-6 and IL-8 mRNA expression levels using real-time RT-PCR. The cell culture supernatants were collected at 48h post-transfection, and protein concentration of secreted IL-6 and IL-8 was determined using BioPlex multi-cytokine assay. **(A)** Depicts the effect of siRNA against p38β on NF-κB p65 nuclear translocation. **(B, D)** mRNA expression levels of IL-6 and IL-8 calculated relative to mock-transfected controls after siRNA mediated silencing, respectively. **(C, E)** Protein concentrations for secreted IL-6 and IL-8 after siRNA mediated silencing, respectively. **(F, G)** Depicts the effect of siRNAs against p38β and p38δ on the expression levels of C/EBP-δ and p-c-jun following Vpr transfection of astrocytes, respectively. Every bar represents the mean ± SE of thee independent experiments done in triplicates. Statistical analyses were performed using 1-way ANOVA using post-hoc Tukey HSD test, **#** p < 0.01, ^**a**^ p < 0.05 as compared to Vpr transfected cells; ** p < 0.01 and * p < 0.05 compared to mock-transfected controls.

**Fig 7:**
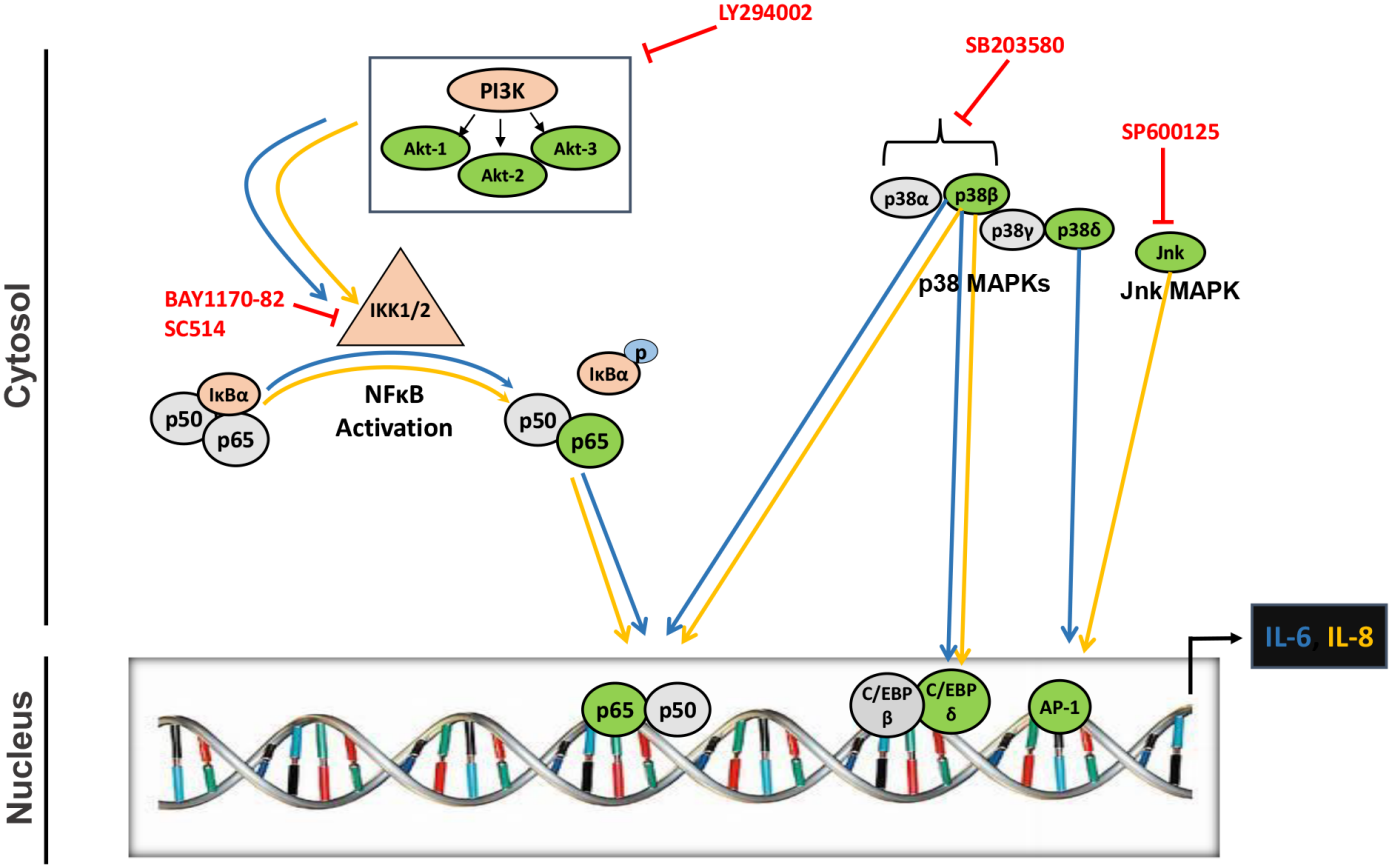
Schematic for the representation of molecular pathways involved in HIV-1 Vpr mediated induction of IL-6 and IL-8 in astrocytes. HIV-1 Vpr activates PI3K/Akt, p38-MAPK and Jnk-MAPK related signaling pathways in SVGA astrocytes. These signaling molecules lead to the activation of transcription factors NF-κB, C/EBP-δ and AP-1, which bind to the IL-6 and IL-8 promoter and induce their expression in astrocytes. *Blue* lines represent pathway for IL-6, while *yellow* lines depict the pathway for IL-8.

Apart from NF-κB, the IL-6 and IL-8 promoters have been shown to contain binding sequences for the transcription factors belonging to AP-1 and C/EBP family. Our results showing abrogation in the levels of IL-6 by p38β and p38δ MAPK; and IL-8 by p38β MAPK raised the possibility of utilization of other transcription factors by HIV-1 Vpr in astrocytes. NF-κB is known to be activated by upstream p38β MAPK. We therefore looked into the effect of p38β MAPK silencing on the nuclear translocation of p65 in response to HIV-1 Vpr in astrocytes. We observed a substantial reduction in the nuclear levels of p65 when p38β MAPK was silenced in HIV-1 Vpr transfected astrocytes as compared to non-silenced cells (Fig 6A). The role of C/EBP-β, C/EBP-δ and AP-1 was dissected by the use of specific siRNA’s directed against each transcription factor. Interestingly, only siRNA’s against C/EBP-δ and AP-1 transcription factor significantly abrogated the mRNA and protein expression levels of IL-6 and IL-8 by HIV-1 Vpr in astrocytes (Fig 6B–6E). The siRNA against C/EBP-β was surprisingly not effective in reducing HIV-1 Vpr mediated induction in IL-6 and IL-8 levels. The involvement of p38β and p38δ in the activation of C/EBP-δ and AP-1 was further confirmed as siRNA mediated silencing of p38β and p38δ significantly reduced the levels of C/EBP-δ and p-c-jun in Vpr transfected astrocytes as compared to non siRNA transfected controls, respectively (Fig 6F and 6G). The schematic representation of molecular mechanisms involved in HIV-1 Vpr mediated induction of IL-6 and IL-8 in astrocytes (Fig 7).

## Discussion

The major obstacle to the successful development of adjunct therapies for the prevention or treatment of HAND remains the incomplete understanding of the molecular mechanisms that are involved in the development of HAND. A plethora of studies have shown the importance of HIV-1 infected monocyte derived macrophage (MDM) and microglia in progression of HAND. However the role of astrocytes in the progression of HAND still remain elusive. Astrocytes act as a reservoir for HIV-1 in the brain and maintain low level of viral replication and activity even when the virus is suppressed by HAART in the periphery [28,29]. This low level of virus replication is sufficient to induce a neuroinflammatory response from astrocytes, that has the potential to enhance viral replication in astrocytes and aggravate inflammation in the brain [8]. The importance of cytokines in this process is underscored by many findings that outline their role in almost every aspect of HIV-1 related neuropathogenesis including the disruption of BBB and neuronal apoptosis [30,31]. Modulating the pathways involved in the upregulation and secretion of cytokines seems a plausible approach to ameliorate the symptoms of HAND and improve quality of life in HIV-1 infected individuals. Our results in the present study illustrate the mechanisms involved in Vpr-induced cytokine expression.

HIV-1 Vpr is present in the blood and cerebrospinal fluid of patients with HIV-1 encephalitis [32]. It is reasonable to assume that extracellular Vpr shedding would be reduced in patients on suppressive HAART but that has not been shown. We were specifically interested in looking at the endogenous HIV-1 Vpr expression. To that end, we transfected an expression plasmid encoding HIV-1 Vpr into the astrocytes and looked into the induction of multiple cytokines that have been shown to be involved in the HIV-1 related neuropathogenesis. We found that the levels of IL-6 and IL-8 were substantially induced by HIV-1 Vpr in astrocytes. We clearly demonstrate that HIV-1 Vpr increases IL-6 and IL-8 in astrocytes at mRNA level as early as 1h post transfection and shows a time-dependent expression pattern. The cell culture supernatants showed significant presence of secreted IL-6 and IL-8 protein at 6h post transfection and increased until 72h. We further confirmed the secreted cytokines by blocking their secretion through the use of golgiplug. IL-6 and IL-8 that were sequestered in the cytosol were immunostained and quantified.

In the following studies, we looked into the mechanisms responsible for the induction of immunoregulatory cytokines IL-6 and IL-8 by HIV-1 Vpr in the astrocytes. Previous studies have shown the involvement of the major cytokine regulating transcription factor NF-κB to be involved in the induction of IL-6 and IL-8 in a variety of cell types [33,34]. Recently, HIV-1 Vpr was also shown to activate both canonical and non-canonical NF-κB pathway [35]. In fact, NF-κB has been demonstrated to be involved in HIV-1 Vpr mediated increased expression of IL-8 in U937 monocytes [13]. We wanted to check the role for NF-κB in induction of IL-6 and IL-8 by HIV-1 Vpr in astrocytes. We observed significant abrogation in the levels of IL-6 and IL-8 with the use of chemical inhibitors targeting NF-κB pathway. In order to rule out any non-target effects associated with chemical inhibitors, we employed specific siRNAs to silence p50 and p65 subunits of NF-κB pathway. Surprisingly, only the silencing of p65 was able to substantially reduce the levels of IL-6 and IL-8. These results point towards the involvement of p65 homodimers in the induction of IL-6 and IL-8. Studies regarding the roles of NF-κB subunits have unequivocally demonstrated that p65 subunit is responsible for the transactivation of κB containing promoters [26,36]. This could explain why only p65 subunit silencing was effective in significantly suppressing IL-6 and IL-8 expression. By knocking down p50 with siRNA, we did not observe any inhibition on the levels of IL-6, however the levels of IL-8 were actually increased compare to Vpr control. These results could be due to the shown suppressive effect of p50 homodimers on κB promoter, which is reduced by p50 silencing and leading to increased IL-8 expression [26].

Our previous work and many others have shown the involvement of PI3K/Akt pathway in the activation of NF-κB [37,38]. The reduction in the levels of IL-6 and IL-8 in the presence of chemical inhibitor targeting PI3K/Akt (LY294002) pathway demonstrates the involvement of this pathway. It was further demonstrated by showing the reduction in the levels of phosphorylated form of Akt and nuclear translocation of p65 subunit of NF-κB with the pre-treatment of LY294002. Moreover, the reduction in HIV-1 Vpr mediated induction of IL-6 and IL-8 was also seen with specific silencing of Akt isoforms; Akt-1, Akt-2 and Akt-3 with siRNAs. This further confirms the involvement of PI3K/Akt/NF-κB signaling in the increased expression of IL-6 and IL-8 by HIV-1 Vpr in astrocytes.

MAP kinases including p38 and Jnk have been shown in many studies to be playing a role in the immunoregulation and controlling the production and secretion of various cytokines [39]. Our results with SB203580, a chemical inhibitor of p38 MAPK pathway showed the abrogation in the levels of IL-6 and IL-8, while the use of Jnk-MAPK inhibitor SP600124 only led to the reduction in IL-8 but not the IL-6 levels. This is in line with the previous evidence which suggests that MAPK p38 and Jnk can regulate the expression of IL-8 [40]. Jnk-MAPK is responsible for the transcriptional activation of IL-8 promoter via AP-1, while p38-MAPK is responsible for stabilizing and preventing the degradation of IL-8 mRNA. On the contrary, SP600124 actually increased the levels of IL-6 at mRNA and protein. This is consistent with the previous findings which have shown that Jnk deficient mice have higher levels of IL-6 and SP600125 can increase the levels of IL-6[41,42]. We failed to see any inhibition in the IL-6 and IL-8 levels by Erk-MAPK inhibitor UO126. Since it is known that SB203580 inhibits only the α and β isoforms of p38-MAPK but does not affect the γ and δ isoforms [27], we used specific siRNAs to silence p38-MAPK isoforms and look into the effect on HIV-1 Vpr mediated induction of IL-6 and IL-8 in astrocytes. Our results clearly demonstrate that only β isoform of p38-MAPK is involved in upregulation of IL-6 and IL-8, while δ isoform is also responsible for IL-6 induction. Differential expression of p38 isoforms have been previously reported in the ischemic brain models where p38β was prominently activated in astrocytes, while p38α was activated in microglia [43]. Recently, p38δ was also identified as a novel target of ceramide that regulates the biosynthesis of IL-6[44].

The activation of multiple protein kinases by HIV-1 Vpr as well as the presence and importance of other transcription factors present in the IL-6 and IL-8 promoters propelled us to look into the possibility of involvement of other transcription factors [45,46]. As p38β has been shown to activate C/EBP family of transcription factors such as C/EBP-β and C/EBP-δ which are shown to be predominant isoforms in astrocytes with the potential to regulate IL-6 expression [47] we employed specific siRNAs against each of them to check their effect on the induction of IL-6 and IL-8 by HIV-1 Vpr. Interestingly, only silencing of C/EBP-δ was able to significantly reduce the levels of IL-6 and IL-8. This is in line with the studies focusing on the role of glia in neuroinflammation which have also identified C/EBP-δ as an important transcription factor and its knockout attenuated the levels of IL-6 in mice brains [48]. Differential effects of C/EBP-β and C/EBP-δ have also been documented in LPS induced lung inflammation where C/EBP-δ but not C/EBP-β was able to reduce the levels of IL-6 production [49]. Furthermore, C/EBP-δ have been demonstrated to have a stronger transactivation potential than C/EBP-β [50]. Recently, work from our lab and others have identified that p38δ isoform can regulate the transcription factor AP-1 which has also been shown to be activated by Jnk-MAPK and plays an essential role in IL-6 and IL-8 production [46,51]. Our results using siRNA targeting AP-1 clearly demonstrate its involvement in HIV-1 Vpr mediated induction of IL-6 and IL-8. Moreover, we also illustrate that p38δ is responsible for the activation of AP-1 in astrocytes.

Our study points towards the involvement of multiple protein kinases via the activation of transcription factors NF-κB, AP-1 and C/EBP-δ in the regulation of induction of IL-6 and IL-8 by HIV-1 Vpr in astrocytes. All three of these transcription factors can interact with HIV-1 LTR promoter and thus can modulate HIV-1 replication [52,53]. NF-κB and C/EBP have been shown to interact and synergistically induce the production of IL-6 and IL-8 that could lead to neurotoxic effects in the brain [54]. Moreover, NF-κB, AP-1 and C/EBP family members have the potential to synergistically interact and activate HIV-1 promoter that can lead to increased HIV-1 replication and production of neuroinflammation. Dissecting the molecular mechanisms involved in the formation of neuroinflammatory milieu that is characteristic of HAND is a critical step towards identifying targets for the adjunct therapies for HAND.

## Supporting Information

S1 Fig HIV-1 Vpr induces IL-6 and IL-8 in primary human fetal astrocytes. Human fetal astrocytes were subjected to electroporation with HIV-1 Vpr expressing plasmid and seeded in 6 well plates. The cells were harvested after 24h of electroporation and RNA was extracted using RNeasy mini kits. Cell culture supernatants were also collected after 24h and secreted IL-6 and IL-8 were measured using multiple cytokine assay kits. (A, C) Relative mRNA expression levels for IL-6 and IL-8 in different donors (1–6), respectively. (B, D) Protein concentration of secreted IL-6 and IL-8 in different donors (1–6), respectively. Every bar represents mean ± SE of individual donor in triplicates. Statistical analyses were performed using 1-way ANOVA using post-hoc Tukey HSD test, ** p < 0.01 and * p < 0.05.
(JPG)
