# Supplementary figures and images for: Multiple Protein Kinases via Activation of Transcription Factors NF-κB, AP-1 and C/EBP-δ Regulate the IL-6/IL-8 Production by HIV-1 Vpr in Astrocytes
Source: PLoS One. 2015 Aug 13;10(8):e0135633. doi: 10.1371/journal.pone.0135633 (PMC4535882; doi:10.1371/journal.pone.0135633)

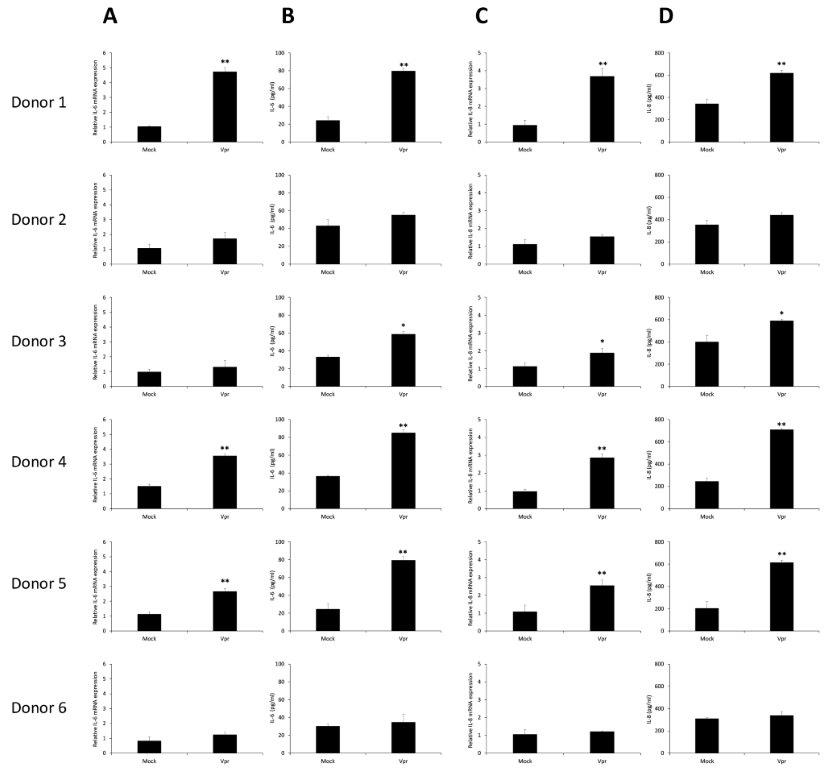

Supplement: S1 Fig — Human fetal astrocytes were subjected to electroporation with HIV-1 Vpr expressing plasmid and seeded in 6 well plates. The cells were harvested after 24h of electroporation and RNA was extracted using RNeasy mini kits. Cell culture supernatants were also collected after 24h and secreted IL-6 and IL-8 were measured using multiple cytokine assay kits. (A, C) Relative mRNA expression levels for IL-6 and IL-8 in different donors (1–6), respectively. (B, D) Protein concentration of secreted IL-6 and IL-8 in different donors (1–6), respectively. Every bar represents mean ± SE of individual donor in triplicates. Statistical analyses were performed using 1-way ANOVA using post-hoc Tukey HSD test, ** p < 0.01 and * p < 0.05. (JPG) [file pone.0135633.s001.jpg]
